# Tuberculose péritonéale: aspect laparoscopique de pelvis gelé

**DOI:** 10.11604/pamj.2016.25.81.10037

**Published:** 2016-10-17

**Authors:** Mehdi Kehila, Mohamed Badis Chanoufi

**Affiliations:** 1 Service C de Gynécologie Obstétrique, Centre de Maternité et de Néonatologie de Tunis, Université Tunis El Manar, Tunisie

**Keywords:** Ascite, tuberculose, laparoscopie, pelvis, Ascites, tuberculosis, laparoscopy, pelvis

## Image en médecine

Il s’agit du cas d’une patiente âgée de 38 ans qui nous a été adressée par l’équipe de gastrologie pour exploration d’une ascite de grande abondance apparue depuis 1 mois. Elle n’a pas d’antécédents pathologiques notables, pas de notion de fièvre au long cours ni de contage tuberculeux. La patiente a 2 enfants vivants accouchés par voie basse et des cycles réguliers de 28 jours. Une échographie abdominale réalisée était normale en dehors de l’épanchement péritonéal de grande abondance. Une ponction d'ascite réalisée a conclu à un exsudat et n'a pas mis en évidence de cellules carcinomateuses. La patiente nous a été adressée pour une exploration coeliocopique. Une open coelioscopie réalisée, a permis d'abord d'évacuer 4 litres de liquide jaune citrin. L'exploration a montré une cavité péritonéale totalement tapissée par de très fines granulations couvrant tout le péritoine pariétal et viscéral. L'exploration de l'étage pelvien a mis en évidence un pelvis totalement gelé avec un utérus et des ovaires fragiles saignant au moindre contact qu'on devinait à leur relief. Les trompes utérines ainsi que le reste des organes pelviens étaient méconnaissables. Les biopsies réalisées ont conclu à une tuberculose péritonéale. La patiente a été mise durant 2 mois, sous l’association de 4 antibiotiques: isoniazide, rifampicine, pyrazinamide et éthambutol; puis durant la deuxième phase de 4 mois sous l’association isoniazide et rifampicine. L'évolution était favorable avec disparition totale de l'ascite au bout de trois semaines de traitement.

**Figure 1:**
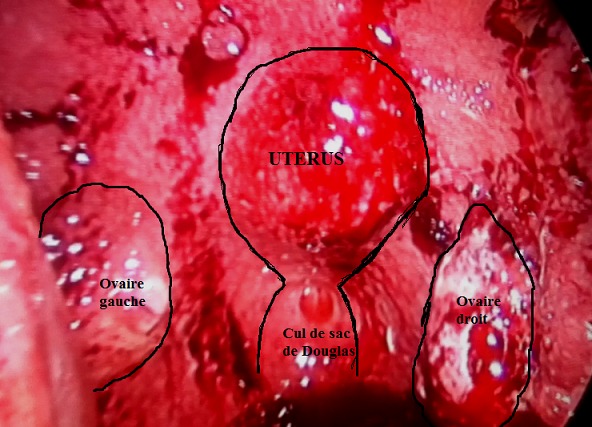
Aspect laparoscopique du pelvis

